# MyoCount: a software tool for the automated quantification of myotube surface area and nuclear fusion index

**DOI:** 10.12688/wellcomeopenres.15055.1

**Published:** 2019-01-21

**Authors:** David P. Murphy, Thomas Nicholson, Simon W. Jones, Mary F. O'Leary

**Affiliations:** 1 Department of Clinical and Movement Neurosciences, Institute of Neurology, University College London, London, UK; 2 Institute of Inflammation and Ageing, MRC-ARUK Centre for Musculoskeletal Ageing Research, University of Birmingham, UK, Birmingham, UK; 3 Department of Sport and Health Sciences, University of Exeter, Exeter, UK

**Keywords:** Image analysis, skeletal muscle, myotube, cell culture

## Abstract

It is often desirable to characterise the morphology of myogenic cultures. To achieve this, the surface area of myotubes is often quantified, along with the nuclear fusion index (NFI). Existing methods of such quantification are time-consuming and subject to error-prone human input. We have developed MyoCount, an open-source program that runs via the freely available MATLAB Runtime and quantifies myotube surface area and NFI. MyoCount allows the user to adjust its parameters to account for differences in image quality, magnification and the colour channels used in generating the image. MyoCount measures of myotube surface area and NFI were compared to the mean of measures performed by two blinded investigators using ImageJ software (surface area R ^2^ = 0.89, NFI R ^2^ =0.87). For NFI, the mean coefficient of variation (CV) between two investigators (17.6 ± 2.3%) was significantly higher than that between the investigator mean and MyoCount (13.5 ± 1.4%). For measurements of myotube area, the CV did not differ between both analysis methods. Given these results and the advantages of applying the same image analysis method uniformly across all images in an experiment, we suggest that MyoCount will be a useful research tool and we publish its source code and instructions for its use alongside this article.

## Introduction

Myogenic cell cultures are commonly used for the investigation of skeletal muscle physiology ^1– 5^. Myotubes formed by such cultures represent useful surrogates for skeletal muscle fibres. Characterising the morphological changes induced by and intervention in myotubes is often of interest and is usually quantified via immunofluorescence-staining of myotubes for a cytoskeletal marker (e.g. desmin or myosin heavy chain) and with DAPI ^6– 9^. The diameter or surface area of myotubes, along with the nuclear fusion index (NFI; defined as the number of nuclei incorporated into myotubes, expressed as a proportion of the total visible nuclei in each field of view) may be measured manually using public-domain (e.g. ImageJ) ^6, 8, 9^ or commercially available (e.g. Photoshop) software ^10^. However, there are issues with such approaches, notably their time-consuming nature and their susceptibility to human error and experimental bias. In human myogenic cultures, we have found that a large number of technical replications are required for each biological replicate to increase the precision of such observations, prior to making comparisons between biological replicates. Thus, the time-consuming nature of such analyses is problematic, as are the subjective decisions that must be made by researchers ( Table 1). The Photoshop method of myotube surface area measurement developed by Agley *et al*. has considerable merits, particularly its ability to quantify the surface area of individual myotubes ^10^. However, it requires the purchase of software and relies upon time-consuming and error-prone manual input.

**Table 1.** MyoCount and the technical challenges associated with myotube morphology analyses.

Problem	Implication for myotube size analysis	MyoCount
Non-cylindrical myotubes	Diameter measurements are inappropriate	Automated surface area measurement.
Nuclear clustering	Human error in accuracy and precision in counting overlapping or clustered nuclei.	While some errors in accuracy may persist, the program applies the same rules to nuclear counting in each image.
Presence of unfused myoblasts in myotube cultures	Myoblast area erroneously included in myotube area measurements. Manual removing myoblasts from analyses is time-consuming and subjective.	MyoCount removes structures containing fewer than 3 nuclei from analyses. The user can further adjust the parameters of analysis.
Large variability in myotube sizes	Averaging myotube sizes may conceal important findings within the myotube size range.	Does not address this issue. Other methods of analysis may be more appropriate if this information is required.
Overlapping myotubes	Difficultly in determining borders of myotubes for analysis	Standardises determination of borders. Like manual counts, some errors persist.

Here, we describe the development of MyoCount ^11^, an open-source program for the automatic quantification of myotube surface area and NFI. The automation of such measurements presents technical challenges ( Table 1). However, we believe that there exist considerable advantages to allowing a computer program to make such judgements consistently between images, rather than relying on the consistency of manual counting by an individual researcher. MyoCount runs via the freely available MATLAB Runtime, and has been designed to be flexible, allowing users to optimise the measurement parameters based on the images available. We anticipate that MyoCount will be useful to researchers requiring a high-throughput method of analysing gross changes in myotube morphology.

## Methods

**Figure 1.**
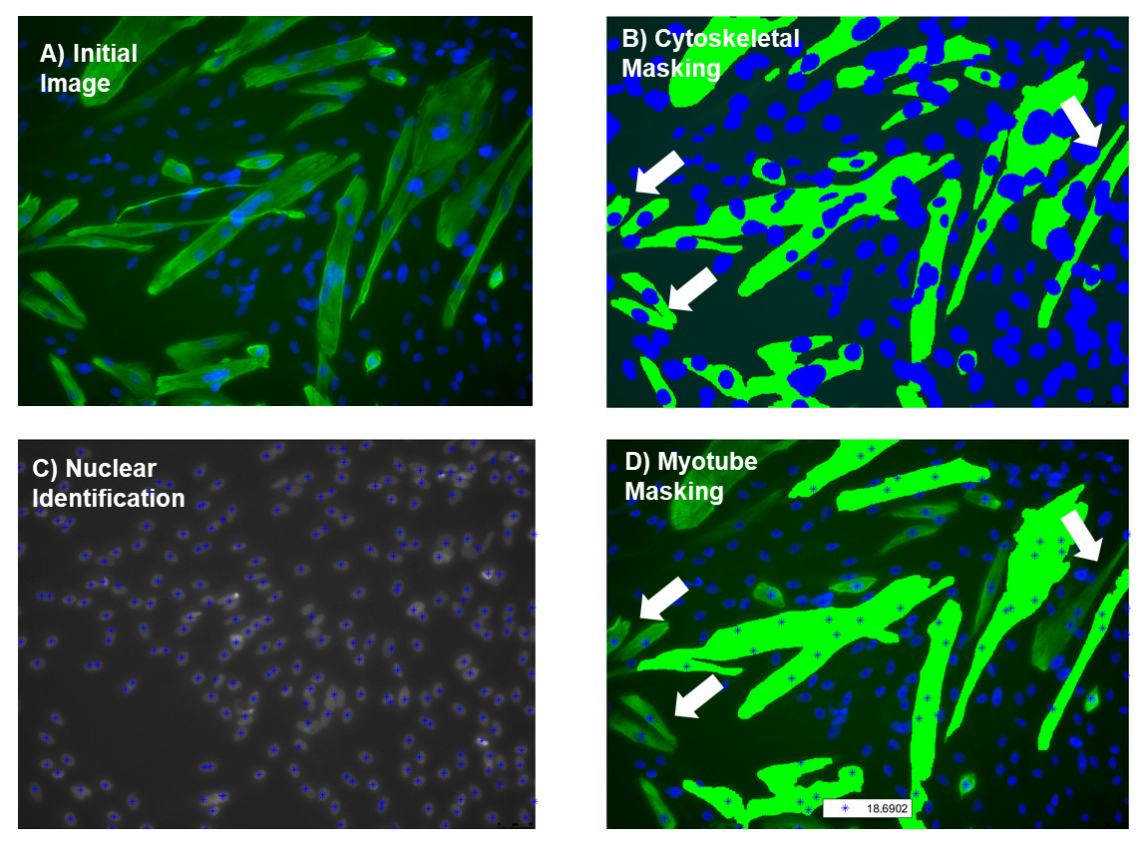
Myoblast exclusion from MyoCount analysis. ( **A**) Original image. ( **B**) MyoCount initially identifies all cytoskeletal areas that exceed the ‘Tube threshold’. ( **C**) Nuclei are identified. ( **D**) Myoblasts (cytoskeletal marker-positive structures containing 2 or fewer nuclei) are eliminated from the analysis. White arrows point to example myoblasts that are removed from analysis.

MyoCount parses tri/bi channel .tif files where one channel is expected to represent myotubes and the other the nuclei. For each input image, the tool produces 4 output images and a summary text file of the results. If running in batch mode (i.e. analysing multiple images at once) it will also generate a summary csv file. The tool identifies myotubes containing at least n nuclei (default setting n = 3) and reports the total number of nuclei in an image, the total nuclei within myotubes and the total nuclei within myotubes containing at least n nuclei. For quality control purposes the tool produces 4 image files comprising an image with myotubes and nuclei highlighted and 3 images highlighting the estimated centroids and approximate borders of the nuclei ( Figure 1).

### Implementation

Myotubes and their approximate borders are identified by normalisation and thresholding to give a binary image. The tool uses filling and smoothing followed by removal of noise—including any objects below the threshold size—to identify approximate borders of individual myotubes. Nuclei and their approximate borders are identified by applying a Wiener filter, thresholding, filling, smoothing and removal of any objects below the threshold size or larger than 1% of the total image.

**Figure 2.**
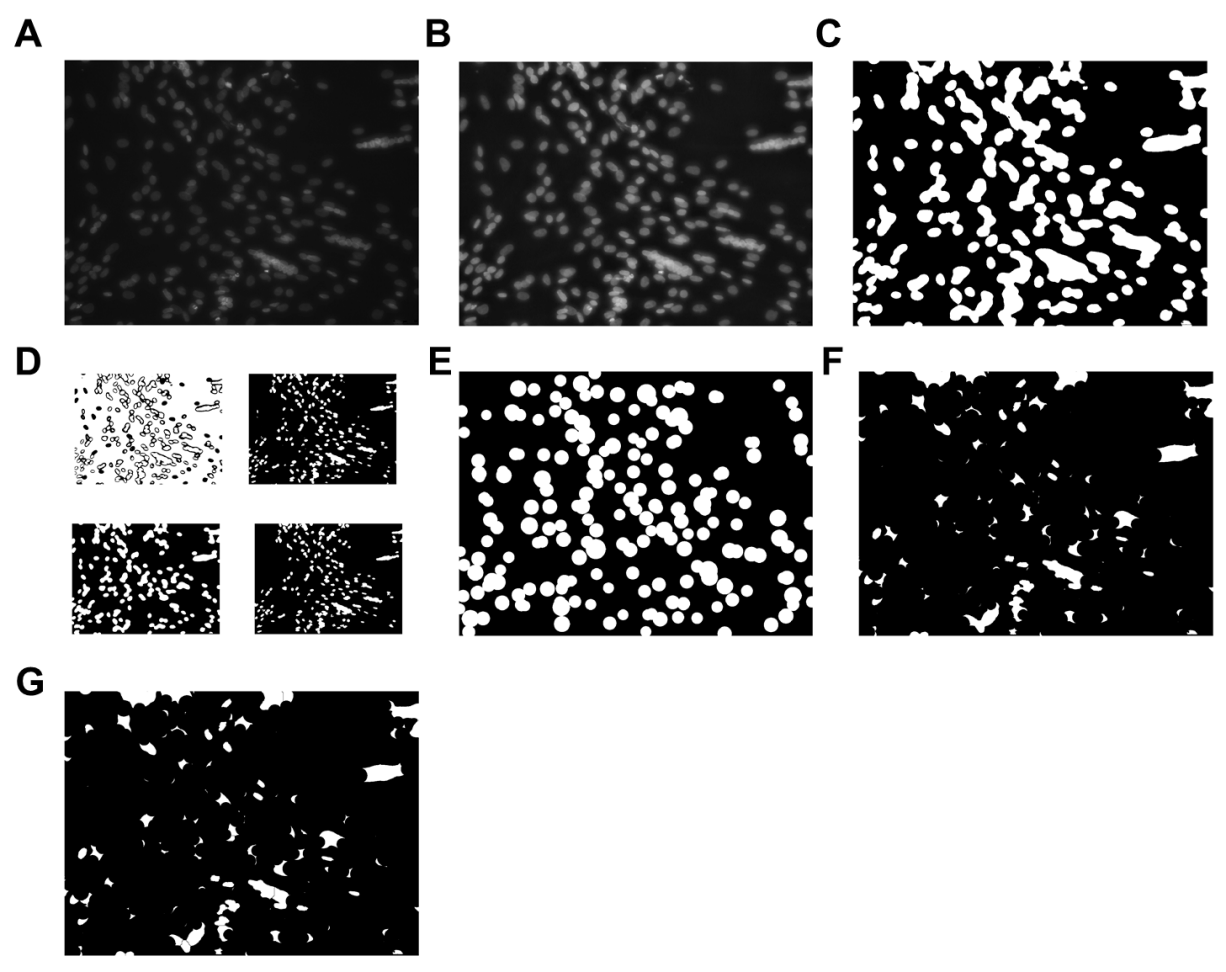
Visual representation of nuclear identification strategy. ( **A**) Original image. ( **B**) Contrast is adjusted and light levels are normalised across the image. ( **C**) Image is binarized. ( **D**) Threshold adjustment. ( **E**) Rounded nuclei are identified. ( **F**) Rounded nuclei are excluded. ( **G**) Remaining DAPI-stained regions are divided into sections that are determined to be of the right size to represent a nucleus. Their centres are identified.

Nuclei are further segmented using a circle Hough transform to identify likely nuclei centre points. Further, watershed segmentation and minima imposition is used on the remaining nuclear regions to identify the remaining centre-points ( Figure 2). The nuclear and myotube overlapping regions are checked and a myotube nuclear count is generated. Some non-peer-reviewed resources are acknowledged as being helpful to us in developing the MyoCount tool ^12– 16^.

### Operation

System requirements: Hardware must be able to run the MATLAB Runtime environment.

### Guidelines for Use

To install MATLAB Runtime:

1. Navigate to http://www.mathworks.com/products/compiler/mcr/index.html.

2. Download the Windows 64-bit version of MATLAB Runtime - version 9.4 (R2018a). 

3. Follow the MATLAB Runtime installation instructions and ensure that you have installed, at lowest, version 9.2 (R2018a). 

To install MyoCount:

1. Navigate to https://github.com/MurphyDavid/MyoCount/releases.

2. Download MyoCount and save to PC.

**Figure 3.**
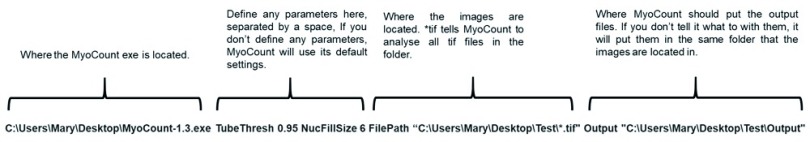
MyoCount command prompt syntax.

To run MyoCount:

MyoCount may be opened directly, by clicking on the application wherever it is saved. The program will prompt you to navigate to an image and will analyse it using the default settings. In order to adjust the parameters of analysis and to run batches of images, we recommend launching MyoCount from the Command Prompt. To launch Command prompt, press the windows + R keys, type ‘cmd’ and press ‘OK’. MyoCount may be launched using the following command line structure - MyoCount1.3.exe [parameter name] [parameter value] ( Figure 3). The MyoCount analysis parameters are outlined in Table 2, along with their default settings.

**Table 2.** MyoCount analysis parameter and their default settings.

Parameter	Default value	Description
SmallestMyotubePixelCount	500	The smallest allowed myotube by the number of pixels it covers.
SmallestNucleusPixelCount	300	The smallest allowed nucleus by the number of pixels it covers.
MyotubeChannel	2	Defines the colour channel for myotubes. The default (2) is the green channel. Red channel = 3.
NucChannel	3	Defines the colour channel for nuclei. The default (1) is the blue channel.
FillSize	10	Chunk size for blurring/smoothing the edges of myotubes.
NucFillSize	5	Chunk size for blurring/smoothing the edges of nuclei.
MinCircleRad	15	The lower bound for nuclear radii. This parameter may need to be adjusted to account for different image magnifications.
MaxCircleRad	40	The upper bound for nuclear radii. This parameter may need to be adjusted to account for different image magnifications.
TubeThresh	1	A lower number will detect fainter cytoskeletal staining, at the expense of sensitivity in distinguishing between proximate myotubes. A higher ‘TubeThresh’ number will limit the detection of cytoskeletal staining to brighter areas but will improve the ability of MyoCount to distinguish between myotubes.
MinNuclei	3	Discard any myotube with fewer than this many recognised nuclei inside its borders.
MaxNucSizeDivisor	100	Discards nuclei larger than 1/n of total image size, where n = the value assigned to the parameter. Can be used to adjust for different image magnifications – 100 works well for 20x images.

### Testing MyoCount

Researchers quantified myotube area in 25 images of immunofluorescence-stained (desmin and DAPI) primary human myotubes using ImageJ (version 1.51o) ^17^. Desmin positive myotubes, containing three or more nuclei were selected using the ‘Freehand selections’ tool, their area was calculated in pixels ^2^ by ImageJ and then converted to μm ^2 ^using a scale embedded in the image. For the MyoCount quantification of myotube area, the program’s default settings were used, with a ‘TubeThresh’ setting of 0.95. The agreement of MyoCount myotube area and NFI measurements with those of two independent, blinded researchers was compared by simple linear regression analysis. The coefficient of variation (CV) between the two researchers and between the mean researcher value and the MyoCount value was calculated for myotube area and NFI in each image. The mean CV for 25 images was calculated. A t-test was used to compare the CV datasets. All data analysis was carried out using GraphPad Prism v5.03 (GraphPad Software, CA, USA). Images used for testing are available on OSF ^18^.

## Results

### Testing the agreement of myocount nuclear fusion index quantification with manual nuclear fusion index quantification

**Figure 4.**
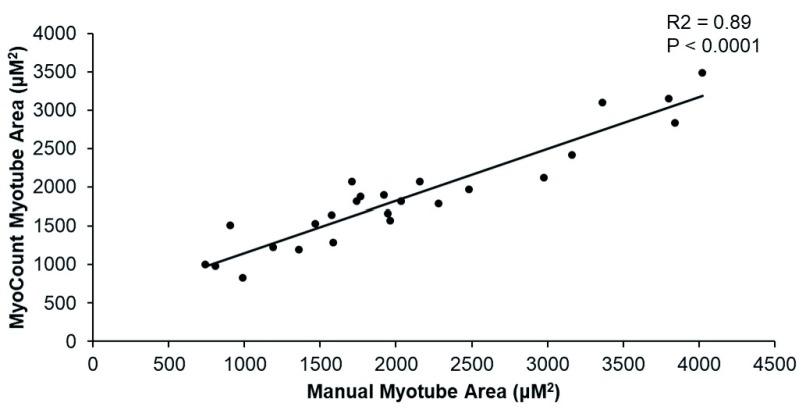
Linear regression for manually calculated myotube area and MyoCount-calculated myotube area.

A simple linear regression was calculated and demonstrated an R ^2^ value of 0.885 (F(1,23) = 177.73, p < 0.0001) ( Figure 4). The mean coefficient of variation between investigators performing manual myotube area quantification was 14.3 ± 2.5% with the CV between the mean investigator myotube area and the MyoCount area being 12 ± 1.7%. This was not significantly different by t-test (p = 0.4).

**Figure 5.**
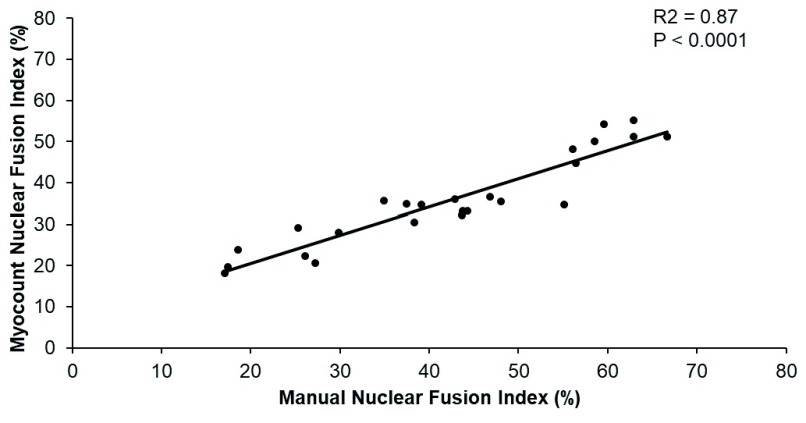
Linear regression for manually calculated nuclear fusion index and MyoCount-calculated nuclear fusion index.

The agreement of MyoCount NFI measurements with those of 2 independent, blinded researchers was quantified. Researchers quantified NFI in 25 images of immunofluorescence stained (desmin and DAPI) primary human myotubes using ImageJ as previously described elsewhere ^6^. MyoCount’s default settings were used, with a ‘TubeThresh’ setting of 0.95. A simple linear regression demonstrated an R ^2^ value of 0.87 (F(1,22) = 145.32, p < 0.0001) ( Figure 5). The mean coefficient of variation between investigators performing manual NFI quantification was 17.6 ± 2.3%, with the CV between the mean investigator NFI and the MyoCount NFI being 13.5 ± 1.4% (p <0.0001 by t test).

## Discussion and conclusions

Here we describe the development of MyoCount ^11^, a software tool for the automated and reproducible quantification of myotube surface area and NFI in immunofluorescence-stained myotubes. MyoCount allows the user to adjust many of its analysis parameters and it produces output files that allow visual verification of such parameter adjustment. As might be expected with complex image analysis, MyoCount is not perfect, and can in certain scenarios, inaccurately identify nuclei or fail to correctly define myotube borders. However, it should be noted that the manual quantification of NFI and myotube surface area is also prone to such inaccuracies. Indeed, for NFI, the mean CV between two investigators for 25 images (17.6 ± 2.3%) was significantly higher than that between the investigators and MyoCount (13.5 ± 1.4%). For measurements of myotube area, the CV did not differ between both analysis methods. MyoCount is advantageous in that it applies the same analysis strategy reliably across all images. The close agreement between the manual and MyoCount quantifications of myotube area and NFI suggest that results generated by MyoCount are faithful to those of manual counts. Given this, and the considerable advantages of MyoCount in terms of eliminating bias and increasing assay throughput, we believe that this version of MyoCount will be of use to the research community.

We anticipate that MyoCount will be useful to researchers looking to quantify substantial changes in myotube morphology between experimental conditions. We acknowledge that manual methods of measuring such outcomes will still play an important role in many research scenarios. MyoCount has been designed to allow its parameters to be adjusted to account for images of different magnifications and images that use different colour channels. It is our hope that users will utilise the open-source code for MyoCount to adapt its functions to suit their research needs and we welcome adjustments and improvements to its function.

## Data availability

The data underlying the results presented in Figure 4 and Figure 5, (.csv and .tif files) are available as ‘Myocount Validation Data’ via OSF. DOI: https://doi.org/10.17605/OSF.IO/F5DXE ^18^.

Data are available under the terms of the Creative Commons Zero "No rights reserved" data waiver (CC0 1.0 Public domain dedication).

## Software availability

Latest source code and Myocount version are available at: https://github.com/MurphyDavid/MyoCount/releases.

Archived source code at time of publication: https://doi.org/10.5281/zenodo.2542811 ^11^.

License: MIT License.
