# Supplementary material for: MyoCount: a software tool for the automated quantification of myotube surface area and nuclear fusion index
Source: Wellcome Open Res. 2019 Jan 21;4:6. [Version 1] doi: 10.12688/wellcomeopenres.15055.1 (PMC6419977; doi:10.12688/wellcomeopenres.15055.1)
Supplement: Peer review history

# Peer review: Reviewer response for version 1

**DOI:** 10.21956/wellcomeopenres.16425.r34789

**Published:** 2019-03-13

**Authors:** Renzhi Han

**Affiliations:** 1 Department of Surgery, Davis Heart and Lung Research Institute, Biomedical Sciences Graduate Program, Ohio State University Wexner Medical Center, Columbus, OH, USA

Quantification of myotube morphology such as surface area and nuclear fusion index is important for muscle biology research. It is often achieved via manual or semi-manual approaches, which are time-consuming and subject to error-prone analyzer bias.

 Here, Murphy *et al.* reported an open-source MATLAB program named MyoCount for automatic quantification of myotube surface area and nuclear fusion index through analyzing immunofluorescence-stained images of myotube cultures. The measurements obtained by MyoCount appear to be in good agreement with those performed by two blinded investigators using ImageJ software, highlighting the suitability of MyoCount to accomplish such time-consuming task.

 The authors should provide sample data files for any researcher who may be interested in testing this program. In Fig. 1, it seems that a large area in the center (closed by three myotubes/myoblasts) was mistakenly identified as one big myotube. Similarly, it appears that several individual myoblasts at the bottom of the image (touching each other) were identified as a myotube. How to minimize such errors? The source code can be annotated more extensively and clearly for users to modify and improve its performance.  

 Minor correction: “induced by and intervention” should be “induced by an intervention”.

I have read this submission. I believe that I have an appropriate level of expertise to confirm that it is of an acceptable scientific standard.

# Peer review: Reviewer response for version 1

**DOI:** 10.21956/wellcomeopenres.16425.r34620

**Published:** 2019-02-15

**Authors:** Kenneth S. Campbell

**Affiliations:** 1 Department of Physiology, University of Kentucky, Lexington, KY, 40536 0298, USA

Analyses of myotube morphology play an important role in current research relating to muscle biology. Most groups implementing these experiments analyze their image data using manual, or in some cases, semi-manual techniques. These are time-consuming and prone to user bias.

 MyoCount, a new open-source program developed by Murphy *et al.*, is a promising new tool that can analyze myotube morphology and nuclear fusion index. It seems likely that MyoCount, or perhaps variants developed from MyoCount, will accelerate research in this area and increase the quality of experimental analyses.

 Murphy *et al.*'s work has many strengths. The manuscript is clearly written and their authors show that the results produced by their software are at least as good, and in some cases, more consistent, than results obtained by manual analysis. Another plus is the fact that the authors have released their source-code and made it freely accessible via the GitHub system.

 Like all new software, the package has some weaknesses. For example, while the code (written in MATLAB) is just ~400 lines long it is only minimally documented. This might make it unnecessarily difficult for researchers to upgrade or enhance the software for future use by the community. The analysis algorithms are also described in minimal detail. For example, the manuscript describes a "TubeThresh" setting when presenting results but it is not entirely clear what this represents until one searches through the code (where it is used with a slightly different name). Perhaps the authors could consider replacing this parameter with an automatic setting (perhaps via Otsu's method or similar) or explaining in the code why this is not appropriate. The software would also have been easier to test if the authors had supplied example data files.

 Despite these minor concerns, MyoCount is a major advance for the field. Developing scientific software is difficult, and making it easy for others to use is harder still. Murphy *et al.* have made an important contribution and the field should be grateful for their efforts.

I have read this submission. I believe that I have an appropriate level of expertise to confirm that it is of an acceptable scientific standard.
